# The *omics* in migraine

**DOI:** 10.1186/1129-2377-14-55

**Published:** 2013-07-01

**Authors:** Luana Lionetto, Giovanna Gentile, Elisa Bellei, Matilde Capi, Donata Sabato, Francesco Marsibilio, Maurizio Simmaco, Luigi Alberto Pini, Paolo Martelletti

**Affiliations:** 1 Sant’Andrea Hospital, Advanced Molecular Diagnostics Unit, Via di Grottarossa 1035 – 1039, Rome 00189, Italy; 2 NESMOS Department, Sapienza University of Rome, Rome, Italy; 3 Department of Diagnostic Medicine, Clinic and Public Health, University of Modena and Reggio Emilia, Modena, Italy; 4 Regional Referral Headache Centre, Sant’Andrea Hospital, Rome, Italy; 5 Inter-Department Headache and Drug Abuse Centre, University of Modena and Reggio Emilia, Modena, Italy; 6 Department of Clinical and Molecular Medicine, Sapienza University of Rome, Rome, Italy

**Keywords:** Genomics, Proteomics, Metabolomics, Migraine

## Abstract

The term *omics* consist of three main areas of molecular biology, such as genomics, proteomics and metabolomics. The *omics* synergism recognise migraine as an ideal study model, due to its multifactorial nature. In this review, the plainly research data featuring in this complex network are reported and analyzed, as single or multiple factor in pathophysiology of migraine. The future of migraine biomolecular research shall be focused on networking among these different and hierarchical disciplines. We have to look for its Ariadne’s tread, in order to see the whole painting of migraine molecular biology.

## Review

### The *omics* synergism

Personalized Medicine aims to evaluate the individual profile of the subject and according to it to proceed to a specific therapeutic strategy.

**Figure 1:**
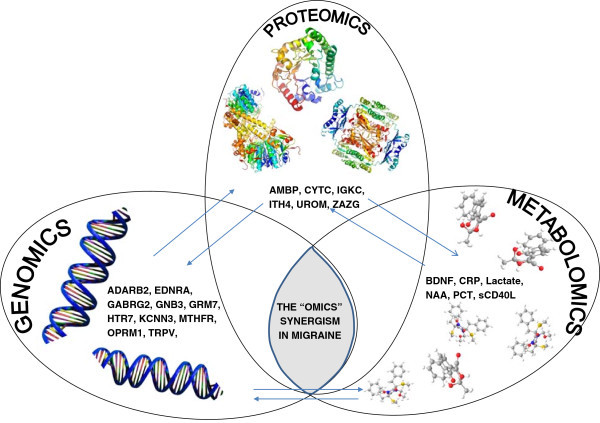
**Framework of the multiple interactions taking place in the migraine *omics *scenario.**

This strategy is made possible by the shift from a hierarchical to a holistic vision of the biological system. In the first case the control of the biological system is generated from the genome up to the lower hierarchical levels represented by proteomics and metabolomics; in the second case, the interactions among these disciplines are evaluated in a synergic way (Figure 1). This new approach is certainly the most appropriate for the understanding of the physiopathology of migraine that, as a multifactorial disease, can only be assessed through the integrated study of the *omics* sciences. Nowadays, it is evident that migraine patients react differently to given drugs, but the exact mechanism has not yet been clarified. In this way, genomic and proteomic research aimed to the identification of molecular pathways involved in drug action is of pivotal importance, in order to focus attention of pharmacogenomics studies on the right targets [1].

### Genomics

In an effort to identify genetic variants involved in migraine risk and influencing the appropriate pharmacological treatments, many genomic studies have been performed in the last years. Due to neurological origin of migraine, some researchers have studied receptors involved in mediation of neuronal activities. Chen et al. [2] characterized one polymorphism in GABRG2 gene encoding the GABAA receptor gamma-2-subunit (rs211037) on a migraine case–control population of 546 subjects. No significant correlation was found. Carreno et al. [3] studied the transient receptor potential (TRP) superfamily of non-selective cationic channels accountable of multimodal sensory and pain perception, central and peripheral sensitization, and regulation of calcium homeostasis, relevant steps of migraine physiopathology. They carried out a migraine-control genetic association study genotyping 149 SNPs covering 14 TRP genes. Consistent results were obtained for TRPV3 rs7217270 in the Migraine with aura group and TRPV1 rs222741 in the overall migraine group, suggesting the involvement of the vanilloid TRPV subfamily of receptors to the genetic susceptibility to migraine [4]. Another gene analyzed is the calcium-activated potassium ion channel gene (KCNN3) involved in neural excitability and in migraine susceptibility. Cox et al. [5] performed a gene-wide SNP genotyping in a high-risk genetic isolate from Norfolk Island, characterized by high percentage of migraineurs. A total of 85 SNPs spanning the KCNN3 gene were genotyped in a sub-sample of 285 related individuals. Only four intronic SNPs displayed gene-wide significance: rs4845663, rs7532286, rs6426929 and rs1218551, with the minor allele in each case conferring protection against migraine risk. Using the same population, Cox et al. [6] carried out another pedigree-based genome-wide association (GWA) study found a significant statistical association with SNP rs4807347 in ZNF555 gene, coding for a zing finger protein. This result has been confirmed in unrelated cohort with more than 500 patients affected by migraine. They also found 4 SNPs in neurotransmitter-related genes (ADARB2, GRM7 and HTR7 genes) suggesting an alteration in the serotoninergic pathway. Maher et al., characterized the entire X chromosome in an association study on the Norfolk population and provides evidence for the SNP rs102834 in the UTR of the HEPH gene, which is involved in iron homeostasis [7,8]. Ligthart et al. [9] performed a meta-analysis of GWA studies for migraine in six population-based European cohorts consisting of 2446 cases and 8534 controls. A total of 32 SNPs showed marginal evidence for association to migraine and the best result was obtained for SNP rs9908234 in the nerve growth factor receptor -NGFR- gene but those results were not replicated in other cohorts. Besides, they found a modest gene-based significant association between migraine and the rs1835740 near the metadherin gene, but further replication studies did not validated this association [10,11].

The opioid system plays an important role in various biological functions including analgesia, drug response and pain reduction. Menon et al. [12] studied the influence of polymorphism in gene coding for the μ-opioid receptor highlighting the association between the OPRM1 A118G SNP and head pain severity in a clinical cohort of 153 female migraineurs with aura. In particular, G118 allele carriers were more likely to be high pain sufferers compared to homozygous carriers of the A118 allele.

Migraine is a complex condition that may in part be related by endothelial and cerebrovascular disruption [13]. Some studies showed that supplementation of B vitamins lowered homocysteine levels and reduced the occurrence of migraine in women [14]. Besides, polymorphisms in genes coding for key player enzymes in the folate metabolic pathway have been investigated in order to define a relation with the pathology and its treatment. The C677T variant in the methylenetetrahydrofolate reductase (MTHFR) has been associated with increased risk of migraine with aura [15,16]. The C allele carrier is also related to higher reduction in homocysteine levels, severity of pain in migraine and percentage of high migraine disability in patient supplemented with B vitamins [14]. The same approach has been adopted for the polymorphism A66G in methionine synthase reductase gene (MTRR): the A allele carriers showed a better response to B vitamin administration [14]. Roecklein et al. [17] performed a haplotype analysis of migraine risk and MTHFR, MTRR and methionine synthase (MTR), including subjects with non-migraine headache (N = 367), migraine without aura (N = 85), migraine with aura (N = 167), and no headache (N = 1347). Haplotype analysis suggested an association between MTRR haplotypes and reduced risk of migraine with aura.

Miao J et al. [18] performed a meta-analysis to determine if polymorphisms in genes linked to the vascular system could be related to migraine. They focused their attention on the receptor for endothelin-1, (EDNRA) known as a potent vasoconstrictor investigating the EDNRA -231G > A SNP. Three studies were included in their meta-analysis for a total of 440 migraineurs, 222 subjects with tension-type headaches (TTHs) and 1323 controls. They found statistical difference between migraineurs and controls with AA genotype vs. AG + GG, suggesting a potential association of -231G > A SNP and migraine. These data suggest that individual SNPs will provide only a small piece of a much larger puzzle composed by a variety of (un) know clinical (phenotypic) information.

Gentile et al. [19] genotyped panel of SNPs involved in triptans pharmacokinetics and pharmacody-namics in a chronic migraine (CM) population. In particular they studied the 30 bp VNTR in MAO A (monoamine oxidase A) promoter, CYP 1A2 *1C and *1 F, CYP3A4 *1B and C825T SNP in the gene coding the G protein b3 subunit (GNB3). A significant association with CM was found for the long allele of monoamine oxidase A 30 bp VNTR and CYP1A2*1 F variant. The same authors performed an analysis of the association between genotypic and allelic frequencies of the analyzed SNPs and the grade of response to triptan administration: a significant correlation for MAOA uVNTR polymorphism was found. Further stratification of patients in abuser and non-abuser groups revealed a significant association with triptan overuse and, within the abusers, with drug response to the CYP1A2*1 F variant [20].

### Proteomics

With the completion of the human genome project in 2002, it has become clear that organism complexity is generated more by a complex proteome than by a complex genome.

Actually, the term “clinical proteomics” defines proteomic technologies employed to examine clinical samples [21]. The available genomic data have now been translated to proteomics, making the discovery of biomarkers increasingly feasible. With its high-throughput and unbiased approach to the analysis of variation in protein expression patterns (actual phenotypic expression of genetic variation), proteomics promises to be the most suitable platform for biomarker discovery. This hopefulness is based on the increasing advances and ability of proteomic technologies to identify thousands of proteins and peptides in complex biological tissues and biofluids, such as plasma, serum and urine [22]. In the past decade, many novel proteomic technologies were emerged and applied to several biological systems for the understanding of cellular activities, disease development and physiological responses to therapeutic interventions and environmental perturbations [23]. One of such emerging field is the application of proteomic technologies to toxicological research, which has given rise to a new area called toxicoproteomics [24]. Drug induced toxicity represents a significant problem in healthcare delivery, therefore the early detection of organ toxicity may provide great benefits to patients, preventing further onset of adverse events and complications of disease management. Incidences of toxicity in liver, heart, brain, kidney and other organs have been reported with the use of different therapeutic drugs. In particular, various epidemiologic investigations proved that different drug types could cause nephrotoxicity, especially in chronic patients.

In this regard, Bellei and co-authors reported two proteomic studies in which they analysed the urinary proteome of medication-overuse headache (MOH) patients, in comparison with healthy non-abuser individuals as control, with the purpose to identify possible differences in excreted proteins induced by the excessive consumption of antimigraine drugs, that could lead to nephrotoxicity [25,26]. Comparing the proteomic profiles of patients and controls, they found a significantly different protein expression at various MW levels, especially in the NSAIDs group, in which six proteins over-secreted from kidney were strongly correlated with various forms of kidney disorders: uromodulin (UROM), alpha-1-microglobulin (AMBP), zinc-alpha-2-glycoprotein (ZAZG), cystatin C (CYTC), Ig-kappa-chain (IGKC), and inter-alpha-trypsin heavy chain H4 (ITIH4). These preliminary results have allowed to define the urinary protein pattern of MOH patients, that was found to be correlated to the abused drug. While adverse effects from long-term triptans use are unknown, the overuse of analgesics may cause well-known unwanted events, including liver dysfunction, gastrointestinal bleeding, addiction and renal insufficiency [27]. Moreover, a recent review concerning the epidemiology of drug-induced disorders has demonstrated that medication overuse could lead to nephrotoxicity and potential renal damage [28]. Currently, the most pertinent application in nephrotoxicology is the proteomic analysis of urine. In recent years, it has proposed a broad range of urinary enzymes and proteins as possible early biomarkers of drug-induced nephrotoxicity [29-31].

### Metabolomics

While the genomics and proteomics suggest a possible mode of operation of the system, metabolomics gives the actual representation of the system. In agreement with the multifactorial nature of the disease, the studies conducted to date relate to different classes of analytes. In fact, several different mechanisms, such as neuro-inflammatory, neurological and cerebrovascular, have so far been suggested in migraine pathophysiology [32].

Whereas the presence of inflammatory processes during migraine attacks, serum levels of different proinflammatory molecules have been investigated. Several studies have found elevated levels of C-Reactive Protein (CRP), a marker of chronic low-grade inflammation, in patients with migraine [33,34]. Also the procalcitonin levels were investigated as indicators of inflammation in migraine patients with and without aura. Turan et al. found significantly high levels of PCT in patients with migraine during the attack compared to the interictal period (0.0485 *vs* 0.0298 ng/ml) [35]. An interesting fact was found by Guldiken et al. about the increase of soluble CD40 ligand (sCD40L). In this study, sCD40L amount was higher in the subgroup of migraineurs with aura than in those without aura and in rare attacks rather than in the frequent ones. In both comparisons the difference in the levels of sCD40L was not significant but these data may be related to the association of migraine with cardiovascular diseases [36].

Also the correlation between migraine and molecules of pain was investigated. Several observation suggested a relationship between low serum levels of vitamin D and higher incidence of chronic pain [37,38]. However, in a large cross-sectional study, Kjaergaard et al. have found that low level of serum vitamin D were associated with non-migraine type of headache [39]. Further studies should be conducted to clarify the potential association of vitamin D and migraine pain. Brain-derived neurotrophic factor (BDNF) is a neurotrophin associated with pain modulation and central sensitization [40]. Fisher et al. have demonstrated that in migraineurs, BDNF was significantly elevated during migraine attacks (31.24 ± 9.31 ng/ml) compared with headache-free periods (24.50 ± 9.17 ng/ml), tension-type headache (20.97 ± 2.49 ng/ml) and healthy controls (21.20 ± 5.64 ng/ml) [41]. This study, showing a correlation between migraine and BDNF, supports the hypothesis that BDNF has a role in the pathophysiology of migraine. The results are in agreement with a pilot study of Tanure et al. [42].

However, NMR or mass spectrometry techniques are able to provide, with an acceptable probability, the description of the current biochemical state of an organism. The development of proton magnetic resonance spectroscopy (^1^H-MRS) has been used to assess noninvasively the metabolic status of human brain [43]. Several studies have employed ^1^H-MRS achieving numerous results for metabolites including N-acetylaspartate (NAA), as a marker of neuronal functioning [44], choline (Cho), as a marker for membrane turnover [45], total creatine (tCr) and lactate, for energy metabolism [46], and myo-inositol (a glial marker) [47]. With the exception of NAA, the results obtained for these molecules are heterogeneous and sometimes contradictory. Indeed the levels of NAA are also increased in the serum [48]. In our opinion, to date, it has not been identified molecules that can be used as a marker for migraine.

Undoubtedly, spectroscopy Nuclear Magnetic Resonance and Mass Spectrometry represent the most powerful and most widely used techniques for the simultaneous analysis of different molecules, allowing together with functional genomics and proteomics to open new roads knowledge in the pathophysiology of migraine. We can suppose that also the progression of the disease may be evaluated by a therapeutic metabolite monitoring approach [49].

## Conclusions

A conclusive scene of *omics* network in migraine pathophysiology is still far to be sharpened. We know a lot of genomics, but less of proteomics and metabolomics of migraine. However, there are numerous scientific evidences assigning an increasingly dominant role to external factors, environmental or behavioural, that interact with the pathophysiology of migraine. Although genomics represents the hierarchical basis of migraine mechanism, proteomics and metabolomics mirror its real image. We have to continue to study the connections among *omics* in order to unsnarl the Ariadne’s tread of migraine.

## Abbreviations

SNP: Single nucleotide polymorphism; CM: Chronic migraine; MOH: Medication-overuse headache; MW: Molecular weight; NSAIDs: Nonsteroidal anti-inflammatory drugs; PCT: Procalcitonin; NMR: Nuclear magnetic resonance.

## Competing interests

All authors declared no competing interest related to the content of this manuscript.

## Authors’ contributions

LL, GG, EB contributed equally in the preparation of the manuscript. LAP and PM conceived the study, provided overall and oversaw the implementation of the study. All the authors approved the final draft of the manuscript. No external assistance in selection, writing or reviewing data has been utilized.
